# *Fusobacterium nucleatum* enhances the efficacy of PD-L1 blockade in colorectal cancer

**DOI:** 10.1038/s41392-021-00795-x

**Published:** 2021-11-19

**Authors:** Yaohui Gao, Dexi Bi, Ruting Xie, Man Li, Jing Guo, Hu Liu, Xianling Guo, Juemin Fang, Tingting Ding, Huiyuan Zhu, Yuan Cao, Meichun Xing, Jiayi Zheng, Qing Xu, Qian Xu, Qing Wei, Huanlong Qin

**Affiliations:** 1 Department of Pathology, Shanghai Tenth People’s Hospital Affiliated to Tongji University, 200072 Shanghai, China; 2 Department of Oncology, Shanghai Tenth People’s Hospital Affiliated to Tongji University, 200072 Shanghai, China; 3 Department of Gastrointestinal Surgery, Shanghai Tenth People’s Hospital Affiliated to Tongji University, 200072 Shanghai, China

**Keywords:** Gastrointestinal cancer, Prognostic markers

## Abstract

Given that only a subset of patients with colorectal cancer (CRC) benefit from immune checkpoint therapy, efforts are ongoing to identify markers that predict immunotherapeutic response. Increasing evidence suggests that microbes influence the efficacy of cancer therapies. *Fusobacterium nucleatum* induces different immune responses in CRC with different microsatellite-instability (MSI) statuses. Here, we investigated the effect of *F. nucleatum* on anti-PD-L1 therapy in CRC. We found that high *F. nucleatum* levels correlate with improved therapeutic responses to PD-1 blockade in patients with CRC. Additionally, *F. nucleatum* enhanced the antitumor effects of PD-L1 blockade on CRC in mice and prolonged survival. Combining *F. nucleatum* supplementation with immunotherapy rescued the therapeutic effects of PD-L1 blockade. Furthermore, *F. nucleatum* induced PD-L1 expression by activating STING signaling and increased the accumulation of interferon-gamma (IFN-γ)^+^ CD8^+^ tumor-infiltrating lymphocytes (TILs) during treatment with PD-L1 blockade, thereby augmenting tumor sensitivity to PD-L1 blockade. Finally, patient-derived organoid models demonstrated that increased *F. nucleatum* levels correlated with an improved therapeutic response to PD-L1 blockade. These findings suggest that *F. nucleatum* may modulate immune checkpoint therapy for CRC.

## Introduction

Immunotherapy has been successfully used to treat a variety of hematological and solid metastatic malignancies in the clinic.^1^ Immune checkpoint therapy, which inhibits the interaction between a T-cell inhibitory receptor and its cognate ligand(s) to activate antitumor immune responses, can elicit durable cancer regression and provides a new treatment for cancer.^2^ The most widely used drugs targeting immune checkpoints, such as programmed cell death protein 1 (PD-1) and its ligand PD-L1, is highly effective in a subset of patients with non-small cell lung cancer, advanced melanoma, bladder cancer, or metastatic renal cell carcinoma.^3–6^ However, anti-PD-1/PD-L1 therapy is considered ineffective in most patients with colorectal cancer (CRC), and only those with microsatellite-instability-high (MSI-high) and a high overall mutational burden have been found to be responsive to anti-PD-1/PD-L1 therapy. Further studies presented the response rate was 50%, and the disease control rate was 89% in a cohort of 28 patients with MSI-high tumors.^7^ It remains challenging to identify CRC patients who will respond to anti-PD-1/PD-L1 treatment, which is necessary to improve the efficacy of this treatment.

The gut microbiota plays a key role in mediating tumor responses to chemotherapy and immune checkpoint therapy in different types of cancer.^8–11^ *F. nucleatum* is a Gram-negative anaerobic bacterium mainly inhabiting the oral cavity and gastrointestinal tract in humans. It has long been considered as an etiological agent of periodontal diseases.^12^ Strikingly, its presence is observed also in multiple types of tumor recently, including esophageal squamous cell carcinoma, gastric cancer and CRC.^13–15^ *F. nucleatum* has been shown to accumulate in colorectal adenocarcinoma and CRC tissues and to be associated with colorectal carcinogenesis via activating E-cadherin/β-catenin signaling and Toll-like receptor (TLR) 4 signaling; this bacterium also promotes chemoresistance in CRC in mice by modulating autophagy.^9,15–18^ Moreover, the amount of *F. nucleatum* in CRC tissues is associated with a poor prognosis.^19^ It has been reported that *F. nucleatum* relates to host immune responses differentially by MSI status in CRC.^20^ The presence of *F. nucleatum* is negatively associated with tumor-infiltrating lymphocytes (TILs) in MSI-high tumors, but positively associated with TILs in microsatellite-stable (MSS) tumors.^20^ However, it is unknown whether *F. nucleatum* affects antitumor immunotherapy, specifically the PD-1/PD-L1 blockade, in CRC.

In the current study, we aimed to investigate whether and how *F. nucleatum* affects the immune system and modulates anti-PD-1/PD-L1 treatment responses in CRC. We found that *F. nucleatum* enhanced the antitumor response to PD-1/PD-L1 checkpoint blockade by inducing PD-L1 expression by activating STING signaling and increasing the accumulation of interferon-gamma (IFN-γ)^+^ CD8^+^ TILs during treatment with PD-L1 blockade. Our findings define a new role of *F. nucleatum* in the immunotherapy of CRC and provide a potential biomarker for clinically predicting the therapeutic effect of PD-1/PD-L1 blockade.

## Results

### High *F. nucleatum* levels correlate with improved therapeutic responses to PD-1 blockade in patients with CRC

**Fig. 1:**
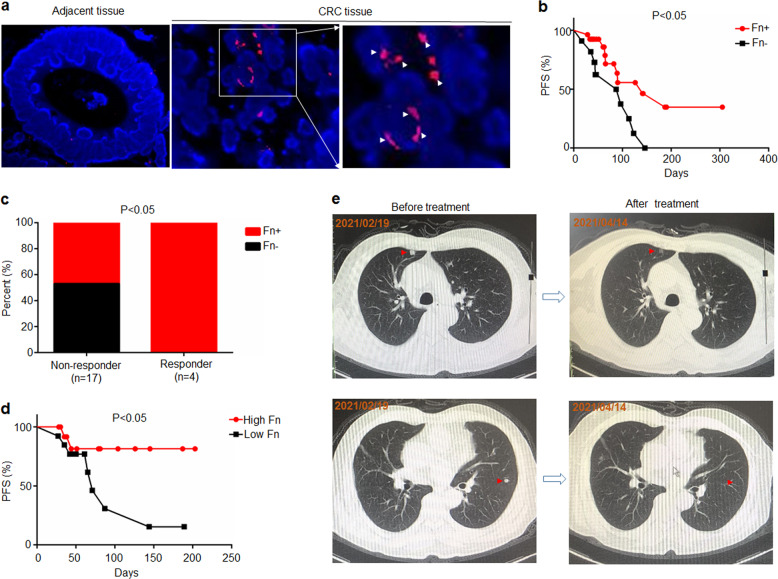
Patients with high levels of *F*. nucleatum were more responsive to PD-1 blockade than those with low levels of *F. nucleatum*. **a** *F. nucleatum* was detected by a fluorescence in situ hybridization (FISH) assay in CRC tumor tissues and adjacent tissues. A FISH assay showed that *F. nucleatum* (red) was present in the tumor tissues of mice. The nuclei (blue) of cells in tumor tissue samples were stained with DAPI. The white arrows indicate positive staining (red) for *F. nucleatum*. **b** The *F. nucleatum* levels in tumor tissues were measured by RT-PCR. The progression-free survival of patients with CRC (*n* = 38) stratified by their *F. nucleatum* levels in tumor tissues. Fn+ and Fn– represent the patients with *F. nucleatum*-positive and negative tumor tissues, respectively. Log-rank test. **c** Correlation analysis between patient outcomes and *F. nucleatum* levels in CRC tumor tissues (*n* = 24). Patients showing no sign of progression within 6 months after PD-1 blockade treatment were defined as responders, while those who progressed within 6 months after PD-1 blockade treatment were defined as non-responder. Chi-square test (one-sided). **d** The progression-free survival of patients with CRC (*n* = 27). The *F. nucleatum* levels in feces were measured by RT-PCR. Patients were divided into high *F. nucleatum* groups and low *F. nucleatum* groups according to the median. Log-rank test. **e** Representative CT images of a patient with lung metastasis before and after treatment of PD-1 blockade. The red arrows indicate lung metastases *nucleatum* were more responsive to PD-1 blockade than those with low levels of *F. nucleatum*

First, we examined whether *F. nucleatum* could affect the response to PD-1/PD-L1 blockade in patients with CRC. We recruited 41 patients with CRC undergoing PD-1 blockade therapy and measured *F. nucleatum* abundance in tumor tissues (Supplementary Table S1). Since some patients with microsatellite-instability-high (MSI-H) or mismatch repair-deficient (dMMR) CRC appear to be susceptible to PD-1/PD-L1 blockade,^21^ we also detected the expression of mismatch repair proteins (MLH1, MSH2, MSH6 and PMS2) in CRC tissues (Supplementary Fig. S1a). We found that the progression-free survival (PFS) of patients with dMMR treated with PD-1 blockade (median 187 days) was longer than that of patients with mismatch repair-proficient (pMMR) (median 89 days) (Supplementary Fig. S1b). The proportion of patients with dMMR CRC who are susceptible to PD-1 blockade (75%) is higher than that of patients with pMMR CRC (11.8%) (Supplementary Fig. S1c). On the other hand, we found that the positive rate of *F. nucleatum* in tumor tissues was 71% (27/38). In addition, *F. nucleatum* is mainly located in CRC tumor tissues rather than adjacent tissues (Fig. 1a). The PFS of patients with *F. nucleatum*-positive tumor tissues (median 141 days) is longer than that of patients with *F. nucleatum*-negative tumor tissues (median 87 days) (Fig. 1b). In addition, the positive rate of *F. nucleatum* in the tumor tissues of responders (100%) is higher than non-responders (47%) (Fig. 1c), suggesting *F. nucleatum* in CRC tissues may be related to the therapeutic effect of PD-1/PD-L1 blockade.

Further, we measured *F. nucleatum* abundance in the feces of patients with CRC (*n* = 27) before the treatment of PD-1 blockade (Supplementary Table S2), found that the positive rate of *F. nucleatum* in the feces of patients with CRC is 100%. We divide patients into high *F. nucleatum* and low *F. nucleatum* groups based on the median. The patients with high *F. nucleatum* levels had longer PFS than those with low *F. nucleatum* levels (Fig. 1d). Interestingly, there is a patient with lung metastasis with high *F. nucleatum* levels in the feces. Although he was in MSS status, the lung metastases became significantly smaller within two months after PD-1 blockade, and the comprehensive assessment reached partial remission (PR) (Fig. 1e). These results indicate that *F. nucleatum* may play a role in anti-PD-1/PD-L1 immunotherapy of patients with CRC.

### *F. nucleatum* augments the antitumor effects of PD-L1 blockade on CRC

**Fig. 2:**
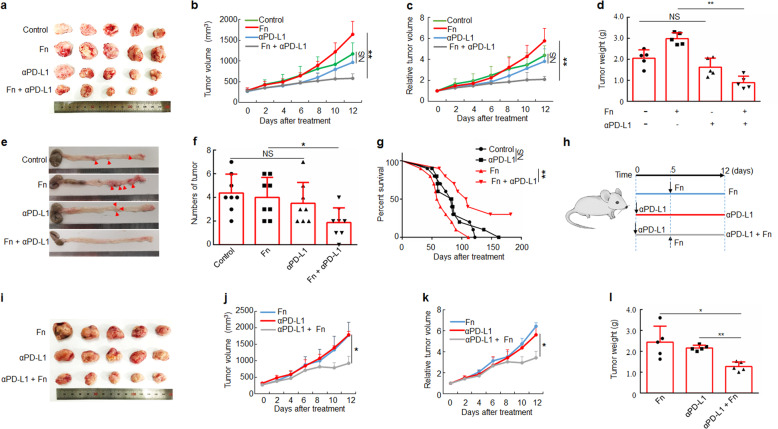
*F. nucleatum* treatment augments the antitumor effects of PD-L1 blockade on CRC. **a**–**d** CT26.WT cells were subcutaneously injected into BALB/c mice. Tumor-bearing mice were intratumorally injected with PBS (control) or *F. nucleatum* and intraperitoneally injected treated with an anti-PD-L1 mAb or an isotype control mAb. **a** CT26.WT tumors from BALB/c mice in different groups at the end of the experiment are shown. Growth curves display tumor volumes **b** and relative tumor volumes **c** over time and the bar graph shows tumor weights at the end of the experiment **d** (One-way analysis of variance [ANOVA] and Bonferroni’s multiple comparison test). **e** Representative image of a colon of AOM/DSS-treated C57BL/6 mice with or without *F nucleatum* treatment and PD-L1 blockade. **f** The tumor numbers (diameter > 1 mm) of the different groups (*n* = 8 per group, One-way ANOVA and Bonferroni’s multiple comparison test). **g** Survival curves of AOM/DSS-treated C57BL/6 mice receiving different treatments (*n* = 10 per group) with Log-rank test. **h** Tumor-bearing mice were first injected intraperitoneally with an anti-PD-L1 mAb or an isotype control mAb and subsequently injected intratumorally with PBS or *F. nucleatum* and continued to be treated with an anti-PD-L1 mAb. **i** A picture of tumors from mice in different groups. **j**–**l** Tumor volume growth, relative tumor volume growth and tumor weights at the end of the experiment are shown. One-way ANOVA and Bonferroni’s multiple comparison test. Data are expressed as the mean + s.d. *P < 0.05, **P < 0.01. NS, no statistical difference. CRC, colorectal cancer. Fn, *F. nucleatum*

We evaluated whether *F. nucleatum* affects the response to PD-L1 blockade in murine models of CRC. Wild-type CT26 mouse colon cancer cells (CT26.WT) were subcutaneously implanted in the flanks of BALB/c mice, followed by intratumoral injection of *F. nucleatum, Escherichia coli* DH-5α or phosphate-buffered saline (PBS) and intraperitoneal injection of an anti-PD-L1 monoclonal antibody (mAb) or an isotype control mAb. Successful colonization of *F. nucleatum* in mouse tumor tissues was observed, and the amount of *F. nucleatum* was similar to that in human CRC specimens (Supplementary Fig. S2). Remarkably, the *F. nucleatum* and PD-L1 blockade co-treatment significantly reduced tumor growth (measured as tumor volume) and tumor weight compared with the treatment with *F. nucleatum* alone (Fig. 2a–d). However, tumor growth and tumor weight did not significantly differ between the control group or the DH-5α injection group and the anti-PD-L1 treatment group (Fig. 2a–d and Supplementary Fig. S3). Thus, *F. nucleatum* injection could enhance the efficacy of anti-PD-L1 treatment in a murine CRC model.

To further examine the role of *F. nucleatum* in the treatment of CRC with PD-L1 blockade, the tumor growth experiment was validated with the bacterium administered via gavage. Thus, we inoculated *F. nucleatum*, DH-5α or PBS into tumor-bearing mice via gavage and then administered an anti-PD-L1 mAb or an isotype control mAb via intraperitoneal injection. As depicted in Supplementary Fig. S4, anti-PD-L1 treatment significantly decreased tumor growth in the *F. nucleatum* treatment group relative to the *F. nucleatum*-only group. However, tumor growth was unaffected by anti-PD-L1 treatment in the PBS control and DH-5α treatment groups. These results confirm that *F. nucleatum* enhances the antitumor effect of the anti-PD-L1 mAb in CRC, and this effect is independent of the routes for *F. nucleatum* administration.

Given its effects on this subcutaneous model, we next examined whether *F. nucleatum* also enhanced the antitumor effects of PD-L1 blockade on a more clinically relevant CRC model. We constructed an AOM/DSS-induced CRC model. We found that the number of tumors (diameter ≥ 1 mm) in *F. nucleatum*-gavage mice was significantly reduced after PD-L1 blockade, while the number of tumors in the control group was not significantly changed after PD-L1 blockade (Fig. 2e, f). In addition, We found that AOM/DSS-treated mice treated with *F. nucleatum* and PD-L1 blockade survived longer than the mice treated with PD-L1 blockade alone (*P* < 0.01) (Fig. 2g). However, in AOM/DSS-treated CRC mice that were not treated with *F. nucleatum*, anti-PD-L1 mAb therapy did not significantly affect survival (*P* > 0.05) (Fig. 2g). The results showed that the presence of *F. nucleatum* increased the survival benefit of PD-L1 blockade in AOM/DSS-treated mice. Collectively, our data indicate that *F. nucleatum* treatment enhances the efficacy of PD-L1 blockade, thereby prolonging the survival time of mice in murine CRC models.

We next investigated whether the therapeutic effect of the anti-PD-L1 mAb could be improved by supplementation with *F. nucleatum*. The anti-PD-L1 mAb was administered into CRC tumor-bearing mice for 5 days. Tumor growth was not found to be inhibited in the anti-PD-L1 mAb group compared with the untreated group. In the following days, those mice were injected intratumorally with *F. nucleatum* (100 µl of a 10^10^ CFU/mL suspension) and the treatment with anti-PD-L1 mAb continued (Fig. 2h). Tumor growth was significantly inhibited after *F. nucleatum* inoculation in mice receiving anti-PD-L1 mAb compared to those treated only with anti-PD-L1 mAb or *F. nucleatum* (Fig. 2i–l). These data indicate that *F. nucleatum* supplementation enhances the therapeutic effect of CRC tumor-bearing mice, which initially do not respond to PD-L1 blockade.

### *F. nucleatum* treatment increases the accumulation of IFN-γ^+^ CD8^+^ TILs during treatment with PD-L1 blockade

**Fig. 3:**
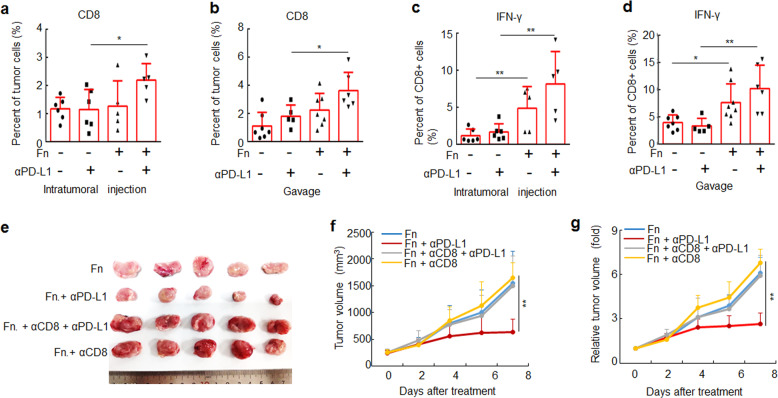
*F. nucleatum* increases the accumulation of CD8^+^ IFN-γ^+^ TILs during treatment with an anti-PD-L1 mAb. **a**, **b** CT26.WT cells were subcutaneously injected into BALB/c mice. Tumor-bearing mice were treated with PBS or *F. nucleatum* by intratumoral injection and intraperitoneally injected with an anti-PD-L1 mAb or an isotype control mAb. **a**, **c** Flow cytometry was used to detect CD8^+^ cells in tumor tissues from mice and the proportion of IFN-γ^+^ cells in CD8^+^ cells. One-way ANOVA and Bonferroni’s multiple comparison test. **b**–**d** CT26.WT cells were subcutaneously injected into BALB/c mice. Tumor-bearing mice were treated with PBS or *F. nucleatum* by garvage and intraperitoneally injected with an anti-PD-L1 mAb or an isotype control mAb. **c**, **d** Flow cytometry was used to detect CD8^+^ cells in tumor tissues from mice and the proportion of IFN-γ^+^ cells in CD8^+^ cells is shown. One-way ANOVA and Bonferroni’s multiple comparison test. **e** Tumor-bearing mice were intraperitoneally injected with an anti-CD8 mAb or an isotype control mAb, then mice were treated with PBS or *F. nucleatum* by intratumoral injection and intraperitoneally injected with an anti-PD-L1 mAb or an isotype control mAb. An image of tumors collected at the end of the experiment is shown. **f**, **g** Tumor volumes and relative tumor volumes at various time points are shown. One-way ANOVA and Bonferroni’s multiple comparison test. Bars, s.d. *P < 0.05; **P < 0.01. Fn, *F. nucleatum*

The accumulation and activity of TILs are major factors affecting the outcome of immune checkpoint therapy, with effector TILs generally promoting antitumor immune response while regulatory populations create an immunosuppressive environment that limits the efficacy.^22,23^ Therefore, we tested how *F. nucleatum*, alone or in combination with anti-PD-L1 mAb treatment, affects TILs. Tumor-bearing mice were treated with *F. nucleatum* by intratumoral injection or gavage and intraperitoneally injected with an anti-PD-L1 mAb. *F. nucleatum* treatment alone did not modulate the proportions of CD4^+^ and CD8^+^ TILs, regardless of *F. nucleatum* administration routes (Fig. 3a, b and Supplementary Fig. S5a, d). However, when combining with anti-PD-L1 mAb treatment, *F. nucleatum* administration led to a significant increase in the proportion of CD8^+^ TILs (Fig. 3a, b). We did not observe any difference in the proportion of regulatory T cells (using FOXP3 as the marker) in tumors among different treatment groups (Supplementary Fig. S5b, c, e, f). In contrast, the DH-5α that was used as a control, did not affect the proportions of CD4^+^, CD8^+^ and FOXP3^+^ TILs during anti-PD-L1 mAb treatment (Supplementary Fig. S6a, b). These results suggest that *F. nucleatum* supplementation increases the proportion of CD8^+^ TILs in mice treated with an anti-PD-L1 mAb, which suggests *F. nucleatum* may enhance the therapeutic effect of anti-PD-L1 mAb by increasing CD8^+^ TILs.

Within the CD8^+^ TIL population, IFN-γ-producing cells have crucial effector roles in antitumor immunity.^22,24^ Therefore, we evaluated the accumulation of IFN-γ^+^ CD8^+^ TILs in the tumor tissues of tumor-bearing BALB/c mice. We observed significant increases in the IFN-γ^+^ CD8^+^ TIL percentage in tumor-bearing mice treated with *F. nucleatum* with or without PD-L1 blockade (Fig. 3c, d). We did not observe any change in the frequency of IFN-γ^+^ CD8^+^ TILs in tumors treated with DH-5α (Supplementary Fig. 6c). Furthermore, antibody-mediated CD8^+^ T-cell depletion significantly diminished the therapeutic effects of the anti-PD-L1 mAb on *F. nucleatum*-supplemented tumor-bearing mice based on tumor volume (Fig. 3e–g). Thus, our results indicate that *F. nucleatum* treatment significantly increases the abundance of IFN-γ^+^ CD8^+^ TILs, which play an important role in the therapeutic effect of *F. nucleatum*-modulated PD-L1 blockade.

### *F. nucleatum* induces PD-1 and PD-L1 expression

**Fig. 4:**
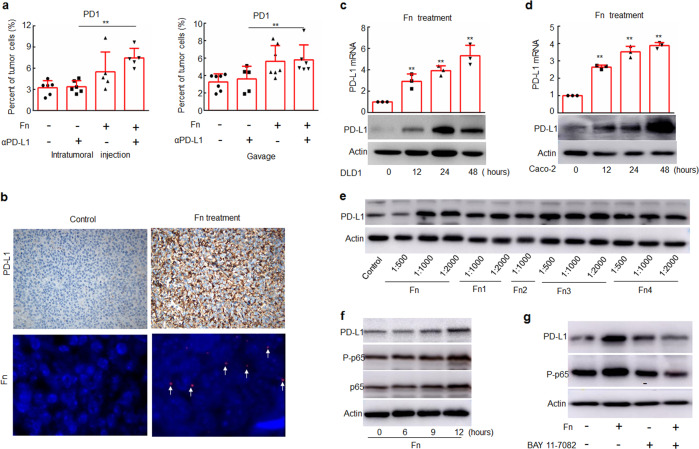
*F. nucleatum* induces the expression of PD-1 and PD-L1. **a** Flow cytometry was used to detect the expression of PD-1 in tumor tissues from mice treated with *F. nucleatum* and/or an anti-PD-L1 mAb. One-way ANOVA and Bonferroni’s multiple comparison test. **b** The protein levels of PD-L1 were detected by IHC, and *F. nucleatum* was detected by FISH in tumor tissue samples from mice. Brown staining in IHC and red staining in FISH (white arrows) indicate positive staining. **c**, **d** DLD1 and Caco-2 cells were treated with Fn (1:1000) for different time course, and the mRNA and protein levels of PD-L1 were detected by RT-PCR and Western blotting, respectively. Student’s *t*-test. **e** DLD1 cells were treated with different dilutions of different Fn isolates obtained from CRC patients. **f** DLD1 cells were treated with Fn (1:1000) for different time course. **g** DLD1 cells were treated with Fn (1:1000) and/or 5 μM BAY 11–7082 for 24 h. The expression of the indicated proteins was detected by Western blotting. Actin was used as a loading control. Bars represent s.d. of at least three experiments. *P < 0.05; **P < 0.01. Fn, *F. nucleatum*. NS, no significant difference

We next examined whether *F. nucleatum* treatment affects responses to anti-PD-L1 mAb treatment by modulating the expression of PD-1 and PD-L1. We found that *F. nucleatum* treatment increased the proportion of PD-1^+^ cells in anti-PD-L1 mAb-treated tumor-bearing BALB/c mice (Fig. 4a). In addition, the expression of PD-L1 was also increased in tumor samples from mice treated with *F. nucleatum* compared with those from mice that did not receive *F. nucleatum* treatment (Fig. 4b and Supplementary Fig. S7). In contrast, DH-5α did not modulate the proportion of PD-1^+^ cells or PD-L1 expression during treatment with the anti-PD-L1 mAb (Supplementary Fig. S8). These results suggest that *F. nucleatum* may enhance the antitumor effect of PD-L1 blockade in murine models of CRC also by modulating PD-1 and PD-L1 expression.

We then used an in vitro system to examine whether and how *F. nucleatum*-modulated PD-L1 expression in human CRC cells. We treated human DLD1 and Caco-2 CRC cells with *F. nucleatum* (1:1000) and found that the bacterial exposure upregulated PD-L1 transcription and protein expression (Fig. 4c, d). Importantly, when DLD1 cells were treated with *F. nucleatum* clinical isolates obtained from CRC patients, PD-L1 expression was also enhanced (Fig. 4e).

We additionally found that *F. nucleatum* activated NF-κB (p65) (Fig. 4f and Supplementary Fig. S9a), one of the most important transcription factors involved in PD-L1 expression.^25^ The *F. nucleatum*-mediated upregulation of PD-L1 expression was abrogated by an NF-κB inhibitor BAY 11–7082 (Fig. 4g and Supplementary Fig. S9b). Thus, it is likely that *F. nucleatum*-modulated PD-L1 transcription by activating NF-κB (p65).

### *F. nucleatum* activates stimulator of interferon genes (STING) signaling in CRC cells

Accumulating evidence has shown that that the activation of STING signaling can activate NF-κB signaling and up-regulate PD-L1 expression.^26,27^ Given the important role of STING signaling in the anti-PD-1/PD-L1 treatment of patients with tumors,^28^ it is no surprise that *F. nucleatum* might activate STING signaling and thereby up-regulate PD-L1 expression by NF-κB, consequently enhancing the therapeutic effect of PD-L1 blockade.

**Fig. 5:**
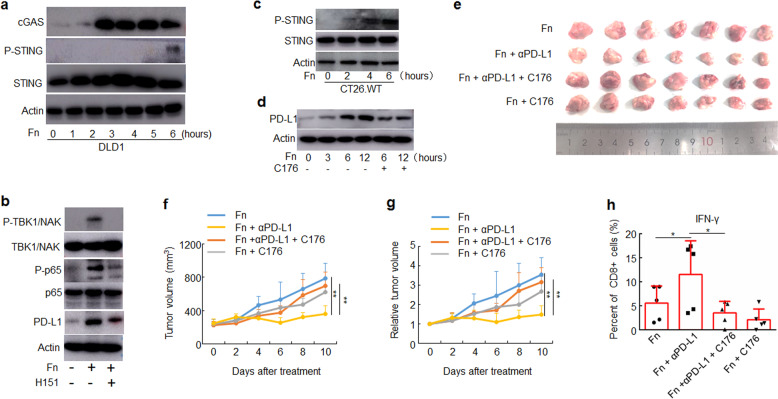
*F. nucleatum* enhanced the therapeutic effect of PD-L1 blockade by activating STING signaling. **a**–**d** The indicated proteins were detected by Western blotting. Actin was used as a loading control. **a** DLD1 cells were treated with Fn (1:1000) for different time course. **b** DLD1 cells were pre-treated with H151 for 2 h, and then treated with Fn (1:1000) for 12 h. **c** CT26.WT cells were treated with Fn (1:1000) for different time course. **d** CT26.WT cells were pre-treated with C176 for 2 h, and then treated with Fn (1:1000) for different time course. **e**–**h** CT26.WT cells were subcutaneously injected into BALB/c mice (*n* = 7 for each group). Tumor-bearing mice were pre-treated with C176 or vehicle, then intratumorally injected with *F. nucleatum* or PBS and intraperitoneally injected treated with an anti-PD-L1 mAb or an isotype control mAb every three days until the end of the experiment. Tumor volumes were measured. **e** An image of tumors collected at the end of the experiment is shown. **f**, **g** Tumor volumes and relative tumor volumes at various time points are shown. One-way ANOVA and Bonferroni’s multiple comparison test. **h** Flow cytometry was used to detect the proportion of IFN-γ^+^ cells in CD8^+^ TILs from mice. One-way ANOVA and Bonferroni’s multiple comparison test. *P < 0.05; **P < 0.01. Fn, *F. nucleatum*

The activation of STING signaling involves the expression of multiple factors including cyclic GMP-AMP (cGAMP) synthase (cGAS), phosphorylation of STING and phosphorylation of tank-binding kinase 1(TBK-1).^28^ To determine whether *F. nucleatum* triggered the activation of STING signaling, we treated human DLD1 cells with *F. nucleatum* (1:1000) and found that the bacterial exposure activated STING signaling by upregulation of cGAS expression and phosphorylation of STING (Fig. 5a). The *F. nucleatum*-mediated activation of NF-κB (p65) and upregulation of PD-L1 expression was abrogated by an inhibitor of human STING signaling H151 (Fig. 5b). In addition, we treated mouse CT26.WT cells with *F. nucleatum* (1:1000) and found that *F. nucleatum* treatment also activated STING signaling (Fig. 5c). The treatment of C176 that is an inhibitor of mouse STING signaling also down-regulated the expression of PD-L1 induced by *F. nucleatum* treatment (Fig. 5d).

In addition, we evaluated whether *F. nucleatum* enhanced the therapeutic effect of PD-L1 blockade by activating the STING signaling in murine models of CRC. CT26.WT were subcutaneously implanted in the flanks of BALB/c mice, followed by C176 treatment and intratumoral injection of *F. nucleatum* and PD-L1 blockade. The *F. nucleatum*-mediated reduction of tumor volume with PD-L1 blockade was abrogated by the C176 (Fig. 5e–g). Moreover, the treatment of C176 abrogated the increasing abundance of IFN-γ^+^ CD8^+^ TILs caused by *F. nucleatum* and PD-L1 blockade treatment (Fig. 5h).

These results suggest that *F. nucleatum* treatment enhances the therapeutic effect of PD-L1 blockade by activating the STING signaling.

### *F. nucleatum* improves therapeutic responses to PD-L1 blockade in CRC organoids

**Fig. 6:**
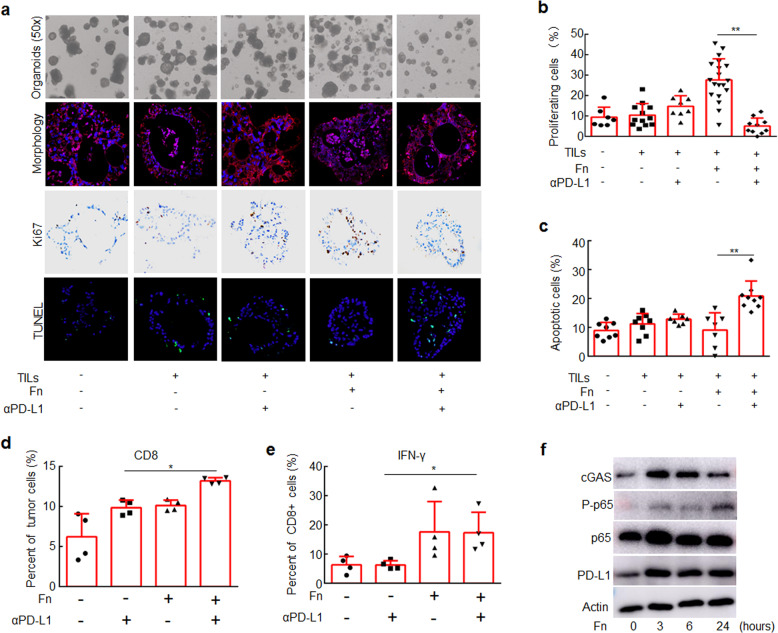
CRC Organoids treated with *F. nucleatum* were more responsive to PD-L1 blockade than those not treated with *F. nucleatum*. CRC organoids were mixed with T lymphocytes (10^5^/well) and *F. nucleatum* (10^8^ CFU) and treated with an anti-PD-L1 mAb or an isotype control mAb for one week. **a** CRC organoids in different groups. Organoid morphology was examined by staining for E-cadherin (red) and with DAPI (blue). Ki-67 expression was detected by IHC (brown staining). Cell apoptosis was detected by TUNEL staining (green). **b** The proportions of proliferating cells were detected by Ki-67 staining. **c** The proportions of apoptotic cells were detected by TUNEL staining. One-way ANOVA and Bonferroni’s multiple comparison test. **d**, **e** Flow cytometry was used to detect the proportion of CD8^+^ cells in CRC organoids and the proportion of IFN-γ^+^ cells in CD8^+^ cells in different groups. One-way ANOVA and Bonferroni’s multiple comparison test. **f** CRC organoids were treated with *F. nucleatum* for different time course. The indicated proteins were detected by Western blotting. One-way ANOVA and Bonferroni’s multiple comparison test. *P < 0.05; **P < 0.01. Fn, *F. nucleatum*

To explore the clinical effects of *F. nucleatum*, we developed an ex vivo model of organoids derived from clinical CRC tissues cultured with TILs derived from the patients and *F. nucleatum* (Supplementary Fig. S10a, b). Tumor cell proliferation was significantly reduced in the organoids treated with *F. nucleatum* and the anti-PD-L1 mAb compared with those treated only with *F. nucleatum* (Fig. 6a, b), and tumor cell apoptosis was significantly increased in *F. nucleatum* and an anti-PD-L1 mAb-treated organoids (Fig. 6a, c and Supplementary Fig. 10c). In contrast, PD-L1 blockade did not significantly affect cell proliferation or apoptosis in organoids that were not treated with *F. nucleatum* (Fig. 6a–c). These results suggest that *F. nucleatum* exposure increases the sensitivity of clinical CRC samples to the antitumor effects of PD-L1 blockade.

In order to further confirm whether the results of organoids are consistent with murine models and cell lines, we detected the changes in CD8^+^ and IFN-γ^+^ CD8^+^ TILs in different groups of organoids. *F. nucleatum* treatment increased the proportion of CD8^+^ and IFN-γ^+^ CD8^+^ TILs in the organoids treated with PD-L1 blockade (Fig. 6d, e). In addition, *F. nucleatum* treatment activated STING signaling by upregulation of cGAS expression and NF-κB signaling by phosphorylation of p65 and increased PD-L1 expression (Fig. 6f).

Taken together, our results show that *F. nucleatum* activates STING signaling and upregulates PD-L1 expression in CRC tumors. During anti-PD-L1 mAb treatment, *F. nucleatum* recruits IFNγ^+^ CD8^+^ TILs to kill tumors.

## Discussion

Increasing evidence suggests that the composition of the host microbiome influences the efficacy of anti-PD-1/PD-L1 therapy.^8,11^ Commensal *Bifidobacterium* augments dendritic cell function, thereby enhancing CD8^+^ T-cell responses and increasing anti-PD-L1 mAb therapeutic efficacy.^10^ *Akkermansia muciniphila* increases the efficacy of PD-1 blockade against epithelial tumors by increasing the recruitment of CCR9^+^ CXCR3^+^ CD4^+^ T cells.^11^ A consortium of 11 bacterial strains elicits CD8^+^ T-cell responses and enhances the therapeutic efficacy of anti-PD-1 therapy.^24^ However, these bacteria rarely colonized the tumor tissues evaluated. We focused on CRC, which is characterized by rich microbial communities, and studied the impact of *F. nucleatum*, which is enriched in CRC tissues, on the effect of PD-L1 blockade. We found that the presence of *F. nucleatum* in CRC tissues and the intestine affected the treatment outcome of anti-PD-L1 therapy via recruitment of IFN-γ^+^ CD8^+^ TILs.

STING is an adaptor protein that induces the secretion of type I interferons and pro-inflammatory cytokines in response to pathogen and host-derived cytosolic DNA.^28^ There is evidence that intratumoral accumulation of gut microbiota facilitates CD47-based immunotherapy via STING signaling.^29^ Given that STING signaling is a mediator in antitumor immune response, the combination treatment with STING agonists and PD-1/PD-L1 blockade induced regression of tumors compared to PD-1/PD-L1 blockade alone.^26,30^ In addition, tumors from mice treated with a STING agonist showed increased PD-L1 expression and IFN-γ^+^ CD8^+^ TILs.^26^ However, little is known about the mechanism of activation of the STING signaling pathway in tumor cells. We have found that *F. nucleatum* can activate the STING signaling in CRC cells, further confirming that activation of the STING signaling in tumor cells can also enhance the therapeutic effect of PD-L1 blockade.

Previous reports revealed that *F. nucleatum* plays detrimental roles in the occurrence and development of CRC by enhancing chemotherapeutic tolerance and suppressing immunity.^9,16,18^ Our results elucidated an alternative role for *F. nucleatum* in improving the therapeutic outcome in CRC. Likewise, a defined commensal consortium that contains another *Fusobacterium*, *Fusobacterium ulcerans*, enhances the therapeutic efficacy of anti-PD-1 therapy by inducing IFN-γ^+^ CD8^+^ T cells. In addition, *Salmonella typhimurium*, another pathogenic bacteria, has been shown to induce potent antitumor effects by activating the immune system.^31–33^ In particular, *Salmonella typhimurium* mutant VNP20009 and its genetically modified strain TAPET-CD have even been applied in clinical trials.^34,35^ These results suggest that some pathogenic bacteria may strengthen the antitumor immune response induced by immunotherapy, including that in the tumor site, under certain conditions. In addition, genetic engineering to modify pathogenic bacteria to enhance the effect of immunotherapy and reduce the pathogenicity of bacteria is worthy of further attention.

In this study, we have found that *F. nucleatum* can activates STING signaling in CRC cells, thereby transcriptionally regulating the expression of PD-L1 through NF-κB. The anti-PD-L1 antibody treatment blocks the binding of PD-1 of TILs and PD-L1 of tumor cells, and further increases IFN-γ^+^ CD8^+^ TILs to kill tumor cells. Our findings provide a potential biomarker for clinically predicting the therapeutic effect of PD-1/PD-L1 blockade.

## Materials and methods

### Cell culture

Human CRC cell lines DLD1 and Caco-2 and mouse colon cancer cell line CT26.WT were purchased from the American Type Culture Collection. Caco-2 and CT26.WT cells were cultured in RPMI-1640 medium (Gibco, Carlsbad, CA) supplemented with 10% fetal bovine serum (FBS). DLD1 cells were cultured in DMEM (Gibco, Carlsbad, CA) supplemented with 10% FBS. Human TILs derived from clinical CRC tissues were cultured in RPMI-1640 medium supplemented with ImmunoCult Hu CD3/CD28 T-Cell Act (10971, STEMCELL), 10 ng/mL IL-2 (abs04045, Absin) and 10% FBS. Mouse TILs derived from mouse CRC tissues were cultured in RPMI-1640 medium supplemented with 0.2 μM anti-Ms-CD3 antibody (1553057, BD Pharmingen), 0.5 μM anti-Ms-CD28 antibody (2553294, BD Pharmingen), 10 ng/mL IL-2 (abs00970, Absin) and 10% FBS. All cells were cultured in a humidified atmosphere with 5% CO_2_ at 37 °C. For the inhibitor-treatment experiments, cells were pre-treated with 5 μM NF-κB inhibitor BAY 11–7082 (Abcam, ab141228) or 1 μM STING inhibitor CSN22907 C176 (CSNpharm, CSN22907) for 2 h and then treated with *F. nucleatum* (1:1000).

### Bacterial culture

The *F. nucleatum* strain ATCC 25586 was obtained from the American Type Culture Collection. *F. nucleatum* was cultured in Columbia blood agar supplemented with 5 mg/mL hemin, 5% defibrinated sheep blood, and 1 mg/mL vitamin K1 (Sigma-Aldrich) in an anaerobic incubator at 37 °C. The *E. coli* strain DH-5α (Tiangen, China) was grown in Luria-Bertani medium with shaking at 37 °C under aerobic conditions (20% O_2_).

### Clinical samples

CRC tumor tissue samples and feces were obtained at Shanghai Tenth People’s Hospital affiliated with Tongji University. For collection of feces s, eligible patients were diagnosed pathologically and clinically as advanced cancer. The patients were treated with an anti-PD-1 mAb alone or in combination with other drugs for at least one course of treatment, and the feces were collected for detection of the *F. nucleatum* content before treatment. For collection of fresh CRC tissues, Patients were pathologically diagnosed with CRC. After surgical resection, the fresh CRC tissues were used to cultivate organoids and isolate TILs. Written informed consent was obtained from all patients, and the study was approved by the Ethical Committee of Shanghai Tenth People’s Hospital (ID Number: SHSY-IEC-4.1/19–180/02) and registered by the Chinese Clinical Trial Registry (ID Number: ChiCTR1900028063).

### Western blotting

Cells were lysed in lysis buffer (2% sodium dodecyl sulfate, 50 mM dithiothreitol, 10% glycerol, and 100 mM Tris-HCl, pH 6.8). The cell lysates were separated by 10% SDS-polyacrylamide gel electrophoresis and transferred to a nitrocellulose membrane (Millipore, USA). Subsequently, the membrane was blocked with 5% fat-free milk or 2% BSA for 1 h and probed successively with a primary antibody overnight at 4 °C and an HRP-conjugated secondary antibody (1:2000, Proteintech) at 37 °C for 1 h. Immunoreactive bands were visualized using HRP Substrate Luminol Reagent (Millipore, USA) and detected using the Amersham Imager 600 (GE, USA). Actin was used as a loading control. The following primary antibodies were used: rabbit polyclonal antibodies against NF-κB p65 (1:1000, Cell Signaling Technology, 8242 s), NF-κB p65 phospho S276 (1:1000, Abcam, ab197426), Phospho-mSTING (1:1000, Cell Signaling Technology, 72971), Phospho-hSTING (1:500, Cell Signaling Technology, 50907), STING (1:1000, Cell Signaling Technology, 13647), TBK1/NAK (1:1000, Cell Signaling Technology, 3504), and Phospho-TBK1/NAK (1:1000, Cell Signaling Technology, 5483), a mouse monoclonal antibody against PD-L1 (1:1000, Abcam, ab210931), and an HRP-conjugated anti-actin mAb (1:2000, Qihe Biological Co., Ltd.).

### Quantitative real-time RT-PCR

Total RNA was obtained with TRIzol Isolation Reagent (Invitrogen), and M-MLV Reverse Transcriptase (Promega) was used to synthesize cDNA. Quantitative real-time PCR analysis of target genes was carried out with Power SYBR Green PCR Master Mix (Applied Biosystems) using an ABI 7500 detection system (Thermo Fisher Scientific). The manufacturer’s general protocol was followed. The following primer pairs were used: PD-L1, 5’-TACTGTCACGGTTCCCAAGG-3’ (forward) and 5’-GGAGAGCTGGTCCTTCAACA-3’ (reverse); and Actin, 5’-CATCCTCACCCTGAAGTACCC-3’ (forward) and 5’-AGCCTGGATAGCAA CGTACATG-3’ (reverse). The 2 (-Delta Delta CT) method was utilized to evaluate gene expression levels. The expression of target genes was normalized to that of actin.

### Assessment of *F. nucleatum* abundance

Genomic DNA was extracted from fresh and frozen clinical feces samples using a fece DNA kit (D4015–01, Omega Bio-tek) and the QIAamp DNA Mini Kit (51304, QIAGEN), respectively. The amplification and detection of *F. nucleatum* DNA were performed using TaqPath^TM^ 1-Step Multiplex Master Mix (1952734, Applied Biosystems) on an Applied Biosystems 7500 detection system. The *PGT* gene was used as a control. The probes and primers (Applied Biosystems) were as follows: FAM probe for *F. nucleatum*, 5’-GTTGACTTTACAGAAGGAGATTA-3’; VIC probe for PGT, 5’-CCATCCATGTCCTCATCTC-3’; *F. nucleatum* primers, 5’-CAACCATTACTTTAACTCTACCATGTTCA-3’ (forward) and 5’-GTTGACTTTACAGAAGGAGATTATGTAAAAATC-3’ (reverse); and PGT primers, 5’-ATCCCCAAAGCACCTGGTTT-3’ (forward) and 5’-AGAGGCCAAGATAGTCCTGGTAA -3’ (reverse). The method for *F. nucleatum* detection was performed as described previously.^18^ For the content of *F. nucleatum* in feces, the median is a cut-off point, which divides patients into high *F. nucleatum* groups and low *F. nucleatum* groups; and for the content of *F. nucleatum* in tumor tissues, no CT value was considered to be Fn-, the others were Fn + .

### Fluorescence in situ hybridization (FISH)

*F. nucleatum* in formalin-fixed paraffin-embedded tissues was detected by FISH, as described previously.^12,36^ The sequence of the Cy3-labeled *F. nucleatum* probe (5’-CGCAATACAGAGTTGAGCCCTGC-3’) was synthesized by Sangon Biotech Company (Shanghai, China).

### Immunohistochemistry (IHC)

IHC was applied to detect protein expression according to a standard protocol.^36^ Immunohistochemical staining was performed with an anti-PD-L1 antibody (1:100, Roche, clone no. SP263) and anti-Ki-67 antibody (1:100, Gene Tech, clone no. SP6). Cells showing membranous staining for PD-L1 and nuclear staining for Ki-67 were considered as positive cells. For the detection of mismatch repair (MMR) proteins, The following primary antibodies were used: MLH1 (1:100, Gene Tech, clone no. GM002), MSH2 (1:100, Gene Tech, clone no. RED2), MSH6 (1:100, Gene Tech, clone no. EP49), and PMS2 (1:100, Gene Tech, clone no. EP51). A negative staining of at least one mismatch repair protein is considered to be dMMR. All staining was blindly scored by two pathologists according to the intensity of staining.

### Mice

For xenograft experiments, 4- to 6-week-old male BALB/c mice were obtained from Shanghai Slark Experimental Animal Co., Ltd. The rodents were supplied with drinking water containing streptomycin (2 mg/mL) and penicillin (2000 U/mL) for 1 week before CT26.WT cells (5 × 10^6^) were subcutaneously injected into the right flank. The mice were randomized into different groups. When the tumor volume reached ~100 mm^3^, 10^9^ colony-forming units (CFU) of *F. nucleatum* or DH-5α or an equal volume of PBS was administered by multipoint intratumoral injection every 3 days until the end of the experiments. Alternatively, another treatment model was established with *F. nucleatum* (10^9^ CFU), DH-5α (10^9^ CFU) or PBS given by gavage. One-hundred micrograms of anti-mouse PD-L1 antibody (BP0101, BioXcell) or an isotype control mAb, which was used as a control, was intraperitoneally administered every 3 days starting on the next day after the first bacterial treatment until the end of the experiment. In studies where CD8^+^ T cells were depleted, 200 μg of anti-mouse CD8 antibody (BE0004–1, BioXcell) was administered by intraperitoneal injection 2 days before treatment with the anti-mPD-L1 antibody and every 3 days thereafter. In studies where STING signaling was inhibited, 1 μM of C176 (CSN22907, CSNpharm) was administered by intraperitoneal injection 1 day before treatment with *F. nucleatum* and every 2 days thereafter. The length and width of tumors were measured every 2 days with a caliper. Tumor volume was calculated with the formula (length × width^2^)/2. Relative tumor volume was calculated by dividing tumor volume on Day X by tumor volume on Day 0.

For AOM/DSS-induced CRC model, 6-week-old male C57BL/6 mice (Shanghai Slark Experimental Animal Co., Ltd) were fed with 2 mg/mL streptomycin and 0.8 mg/mL penicillin in the drinking water for 1 week. The mice were treated with azoxymethane (AOM, 12 mg/kg) by intraperitoneal injection for 1 week followed by 7 successive days of 2% (w/v) DSS in the drinking water, and then were given regular drinking water for 2 weeks. Then the mice were additionally given 2 cycles of 2% DSS in the drinking water for 1 week and regular drinking water for 2 weeks. *F. nucleatum* (2 × 10^9^ CFU) or PBS was administered to the mice by gavage every two days until the end of the experiment. An anti-mPD-L1 mAb (100 mg) or an isotype control mAb was administered by intraperitoneal injection once every 3 days for a total of 12 days beginning *F. nucleatum* treatment for 1 week. Then the mice were sacrificed and the number of tumors was counted.

Mouse experiments were performed in strict accordance with the National Institutes of Health Guidelines for the Care and Use of Laboratory Animals and approved by the Experimental Animal Ethical Committee of Shanghai Tenth People’s Hospital (ID Number: SHSY-2018–3566).

### Patient-derived organoids (PDOs)

Biopsies from CRC patients were collected in 5 mL of PBS containing penicillin-streptomycin on ice. After gently washing and mincing the tissue samples into ~1–2 mm^3^ pieces, the samples were digested with 10 mL of gentle cell dissociation reagent (07174, STEMCELL) on ice on a rocking platform for 30 min. The dissociated cells were passed through a 100-μm cell strainer, pelleted and suspended in ice-cold PBS. The centrifuged cells were then resuspended in growth factor reduced (GFR) Matrigel (356231, Corning) and seeded in a 24-well flat-bottom cell culture plate (3548, Corning). Following solidification at 37 °C in a 5% CO_2_ incubator for 30 min, 300 μL of human IntestiCult™ Organoid Growth Medium (06010, STEMCELL; supplemented with Y27632 (72304, STEMCELL) for the primary culture) was overlaid in the wells coated with Matrigel, and the culture medium was replaced every two days. To passage PDOs, organoids were harvested with ice-cold PBS and were dissociated with mechanical force by pipetting with a 1-mL pipette (160 times per well). The dissociated PDOs were pelleted and washed with ice-cold PBS. The dissociated cells were mixed with human TILs (10^5^/well) and *F. nucleatum* (10^8^ CFU) according to the experimental design. The mixtures were resuspended in GFR Matrigel and cultured in organoid growth medium supplemented with ImmunoCult Hu CD3/CD28 T-Cell Act (10971, STEMCELL), 10 ng/mL IL-2 (abs04045, Absin) and 10% FBS. Ten micrograms of anti-PD-L1 mAb (Roche, clone no. SP263) or isotype control mAb was added to the cell culture plate after 48 h, and the mixtures were incubated for 1 week.

### Acquisition of TILs

Fresh CRC tumor tissue samples from clinical surgery were cut into small pieces and treated with a human tumor dissociation kit (130–096–929, Miltenyi Biotec). Single-cell suspensions were obtained with a Gentle MACS Octo 8 (Miltenyi Biotec) and subsequently filtered through 70-µm cell strainers (BD Pharmingen). Single-cell suspensions were treated with the Human Pan T-cell Isolation kit (130096535, MACS) to obtain TILs, according to the manufacturer’s instructions.

### Cell apoptosis assay

The cell apoptosis of organoids was detected with the In Situ Cell Death Detection Kit, Fluorescein (11684795910, Roche, Germany). Briefly, organoids were centrifuged at 300 rpm, and the pellets were embedded in paraffin. The paraffin-embedded sections of organoids were dewaxed, rehydrated, treated with Proteinase K (V900887, Sigma), and permeabilized. After labeling with a TdT-mediated dUTP Nick-End Labeling (TUNEL) reaction mixture according to the manufacturer’s instructions, slides were analyzed with an Olympus BX51 fluorescence microscope. TUNELs BX51 fluoresce (green) and total cells (blue) in five regions of the slide were quantified. The proportion of apoptotic cells was calculated by the formula (Number of TUNEL-positive cells/Number of total cells).

### Flow cytometry analyses

Mouse tumor samples were cut into small pieces and treated with a mouse tumor dissociation kit (130–096–730, Miltenyi Biotec). Single-cell suspensions were obtained with a Gentle MACS Octo 8 (Miltenyi Biotec) and subsequently filtered through 70-µm cell strainers (BD Pharmingen). For intracellular IFN-γ detection, cells were pre-stimulated with Leukocyte Activation Cocktail with BD GolgiPlug (550583, BD Pharmingen) at 37 °C for 4–6 h. Dead cells were excluded using FVD506 (65086618, eBioscience). For surface staining, cells (105) were stained with 5 μL anti-CD4, CD8 and PD-1 antibodies in 100 μL of staining buffer for 30 min at room temperature and dark. For intracellular staining, cells were resuspended in 1 mL of Transcription Factor Staining Buffers (5523, eBioscience) and incubated at room temperature for 45 min, then centrifuged to discard the supernatant. One-hundred microliters of Transcription Factor Staining Buffers was added to resuspend the cells, then anti-IFN-γ antibody was added at room temperature Incubate in the dark for 30 min; was added to resuspend the cells (eBioscience, Cat#5523), and then anti-FOXP3 and IFN-γ antibodies were added to the cell suspension and incubated in the dark at room temperature for 30 min. Date was performed on a FACSCanto™ flow cytometer (Becton Dickinson, San Jose, CA, USA) and Flowjo was used to analyze the flow cytometry data. The following antibodies were used: anti-mouse antibodies specific for CD4 (557667 and 557307, BD Pharmingen), CD8 (551162, BD Pharmingen), FOXP3 (560401, BD Pharmingen; 12–5773–82, eBioscience), IFN-γ (FITC and 563376, BD Pharmingen), and PD-1 (551892, BD Pharmingen) and anti-human antibodies specific for CD3 (564713, BD Pharmingen), CD8 (560662, BD Pharmingen), and IFN-γ (562988, BD Pharmingen). The dilution of all antibodies was 1:20.

### Statistical analysis

All statistical analyses were performed with GraphPad Prism 6 software or the statistical package for IBM SPSS Institute 20. Each experiment was repeated at least three times. The *P*-values for comparisons between two groups were obtained with a two-sided Student’s *t*-test. One-way ANOVA followed by a Bonferroni post hoc test was used for multiple comparisons. The Pearson’s *χ*^2^ test was used to evaluate associations. Analyses of overall survival and progression-free survival (PFS) were performed using Kaplan–Meier survival curves and log-rank test. *P* < 0.05 was considered statistically significant (*P < 0.05; **P < 0.01).

## Supplementary information

Supplementary Material

## Data Availability

All data supporting this paper are present within the paper and/or the Supplementary Materials. The original datasets are also available from the corresponding author upon request.
