# Correction To: *Fusobacterium nucleatum* enhances the efficacy of PD-L1 blockade in colorectal cancer

**DOI:** 10.1038/s41392-021-00840-9

**Published:** 2021-12-21

**Authors:** Yaohui Gao, Dexi Bi, Ruting Xie, Man Li, Jing Guo, Hu Liu, Xianling Guo, Juemin Fang, Tingting Ding, Huiyuan Zhu, Yuan Cao, Meichun Xing, Jiayi Zheng, Qing Xu, Qian Xu, Qing Wei, Huanlong Qin

**Affiliations:** 1 Department of Pathology, Shanghai Tenth People’s Hospital Affiliated to Tongji University, 200072 Shanghai, China; 2 Department of Oncology, Shanghai Tenth People’s Hospital Affiliated to Tongji University, 200072 Shanghai, China; 3 Department of Gastrointestinal Surgery, Shanghai Tenth People’s Hospital Affiliated to Tongji University, 200072 Shanghai, China

**Keywords:** Gastrointestinal cancer, Prognostic markers

Correction to: *Signal Transduction and Targeted Therapy* 10.1038/s41392-021-00795-x, published online 19 November 2021

In this article^1^ Qing Wei should have been denoted as a corresponding author along with Huanlong Qin, but was inadvertently removed during the production process.

The original article has been corrected.
