# Supplementary material for: Fusobacterium nucleatum enhances the efficacy of PD-L1 blockade in colorectal cancer
Source: Signal Transduct Target Ther. 2021 Nov 19;6:398. doi: 10.1038/s41392-021-00795-x (PMC8602417; doi:10.1038/s41392-021-00795-x)
Supplement: Supplementary file 1 — Supplementary Material [file 41392_2021_795_MOESM1_ESM.pdf]

Supplementary Materials for

Fusobacterium nucleatum enhances the efficacy of PD-L1 blockade in colorectal cancer

Yaohui Gao[1#], Dexi Bi[1#], Ruting Xie[1#], Man Li[1], Jing Guo[1], Hu Liu[1], Xianling Guo[2], Juemin Fang[2], Tingting Ding[1], Huiyuan Zhu[1], Yuan Cao[1], Meichun Xing[1], Jiayi Zheng[1], Qing Xu[2], Qian Xu[3], Qing Wei[1]*, Huanlong Qin[3]*.

*Correspondence to: Wei-Li Zhao, Email: zhao.weili@yahoo.com, and Meng-Meng Ji, Email: jimengmeng025@163.com.

This PDF file includes:

Figures. S1 to S10

Tables S1 to S2

Gao et al. Supplementary Figure 1

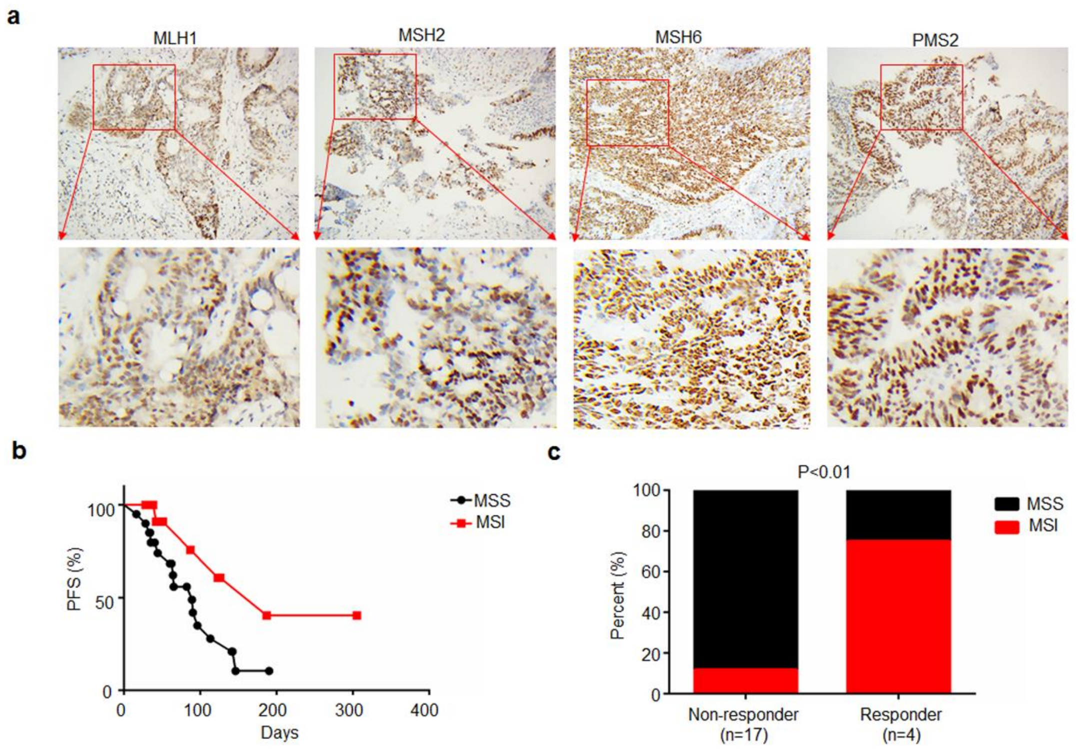

Fig. S1 Patients with dMMR were more responsive to PD-1 blockade than those with pMMR. **a** Representative Immunohistochemistry images of mismatch repair proteins in CRC tumor tissues. **b** The progression-free survival of patients with CRC (n=35). **c** Correlation analysis between patient outcomes and different MMR status. Chi-square test (one-sided).

Gao et al. Supplementary Figure 2

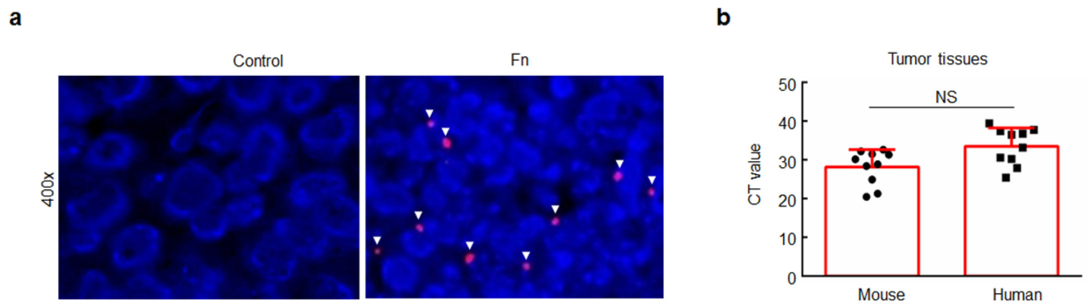

Fig. S2 *F. nucleatum* could colonize the tumor tissue of mice injected intratumorally with *F. nucleatum*. CT26.WT cells were injected subcutaneously into BALB/c mice. Tumor-bearing mice were intratumorally injected with PBS (control) or *F. nucleatum* (10^9 CFU) every two days for two weeks. **a** A FISH assay showed that *F. nucleatum* (red) was present in the tumor tissues of mice. The nuclei (blue) of cells in tumor tissue samples were stained with DAPI. The white arrows indicate positive staining (red) for *F. nucleatum*. The images are representative of the different treatment groups. **b** *F. nucleatum* was detected by RT-PCR in mouse and human CRC tissue samples (n=10). Data are expressed as mean + s.d. Student's t-test. NS, not significant. Fn, *F. nucleatum*.

Gao et al. Supplementary Figure 3

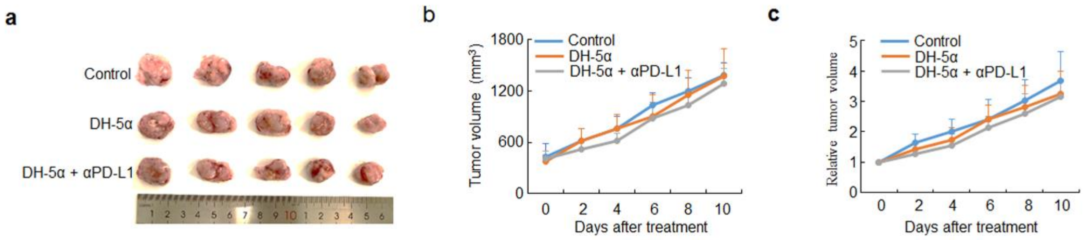

Fig. S3 Intratumoral injection of DH-5α did not affect the therapeutic efficacy of PD-L1 blockade. CT26.WT cells were subcutaneously injected into BALB/c mice. Tumor-bearing mice were intratumorally injected with PBS (control) or DH-5α (10^9 CFU) every two days and were intraperitoneally injected with an anti-PD-L1 mAb or an isotype control mAb. **a** An image of tumors collected in different groups at the end of the experiment is shown. **b, c** Tumor volumes and relative tumor volumes in different groups are shown. Data are shown as mean ± s.d. One-way ANOVA and Bonferroni’s multiple comparison test. Fn, *F. nucleatum*.

Gao et al. Supplementary Figure 4

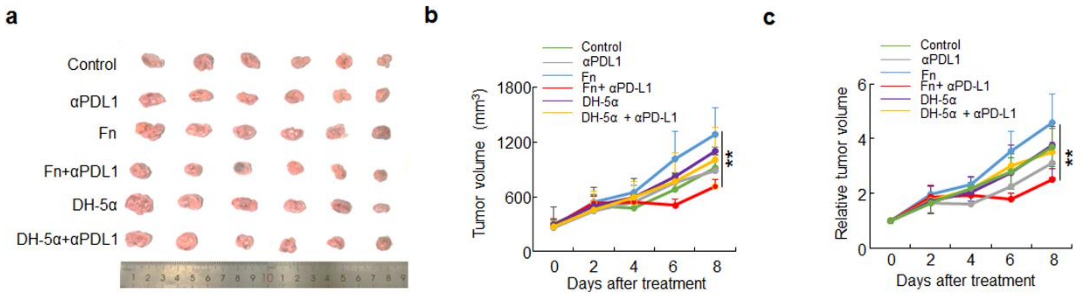

Fig. S4 Gavage administration of *F. nucleatum* augmented the antitumor effects of PD-L1 blockade on CRC. CT26.WT cells were injected subcutaneously into BALB/c mice. Tumor-bearing mice were administered with PBS (control), *F. nucleatum* (10^9 CFU) or DH-5α (10^9 CFU) by gavage every two days and treated with an anti-PD-L1 mAb or an isotype control mAb by intraperitoneal injection. Tumor volumes were measured. **a** A picture of tumors from mice in different groups are shown. **b, c** Tumor volume growth and relative tumor volume growth were assessed. One-way ANOVA and Bonferroni's multiple comparison test. **P<0.01.

Gao et al. Supplementary Figure 5

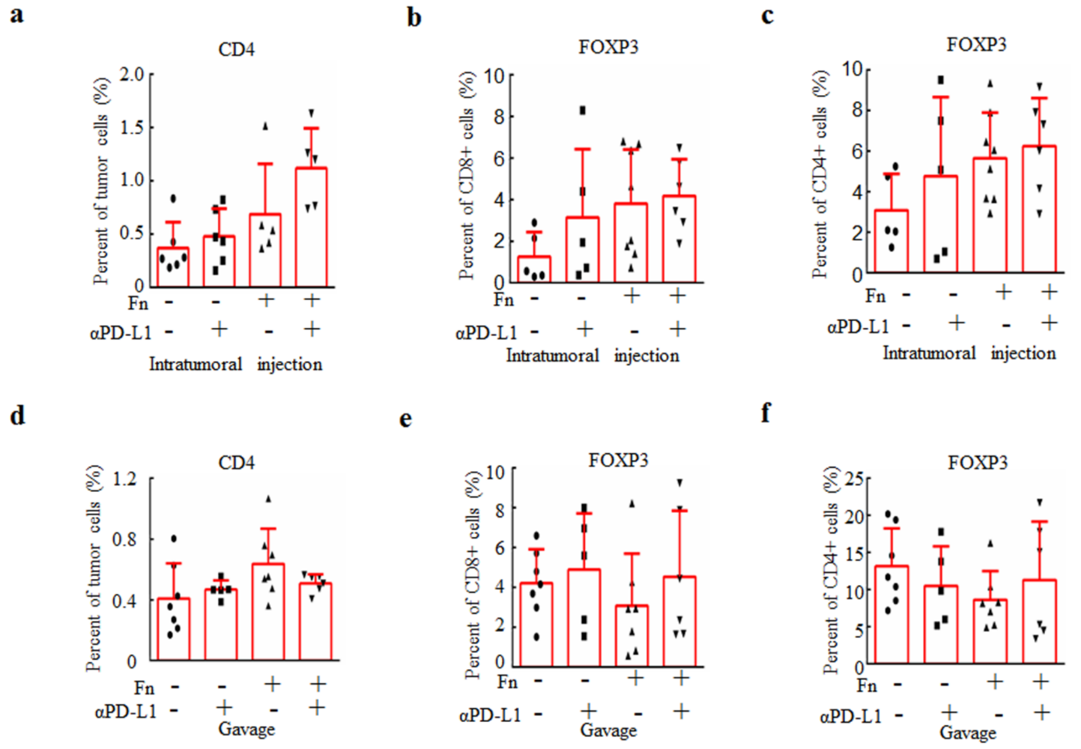

Fig. S5 *F. nucleatum* did not modulate the proportion of CD4^{+} and FOXP3^{+} T cells during treatment with an anti-PD-L1 mAb. CT26.WT cells were subcutaneously injected into BALB/c mice. **a-c** Tumor-bearing mice were treated with PBS or *F. nucleatum* by intratumoral injection and intraperitoneally injected with an anti-PD-L1 mAb or an isotype control mAb. Flow cytometry was used to detect the levels of different types of T lymphocytes in the tumor tissues of mice. One-way ANOVA and Bonferroni's multiple comparison test. **d-f** Tumor-bearing mice were treated with PBS or *F. nucleatum* by garage and intraperitoneally injected with an anti-PD-L1 mAb or an isotype control mAb. Flow cytometry was used to detect the levels of different types of T lymphocytes in the tumor tissues. One-way ANOVA and Bonferroni's multiple comparison test.

Gao et al. Supplementary Figure 6

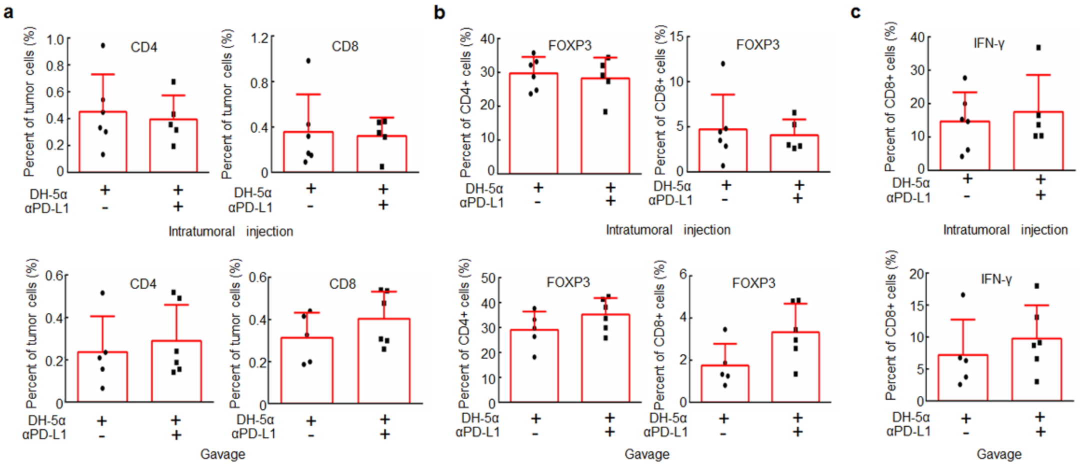

Fig. S6 DH-5α does not modulate the proportion or activity of $CD8^{+}$ T cells during treatment with an anti-PD-L1 mAb. CT26.WT cells were subcutaneously injected into BALB/c mice. Tumor-bearing mice were treated with DH-5α (10^{9} CFU) or PBS (control) by intratumoral injection or garage and intraperitoneally injected with an anti-PD-L1 mAb or an isotype control mAb. For mice treated with DH-5α by intratumoral injection, the anti-PD-L1 mAb treatment time was 9 days. For mice treated with DH-5α by garage, the anti-PD-L1 mAb treatment time is 7 days. **a-c** Flow cytometry was used to detect the levels of different types of T lymphocytes in the tumor tissue of mice. One-way ANOVA and Bonferroni's multiple comparison test. All P>0.05.

Gao et al. Supplementary Figure 7

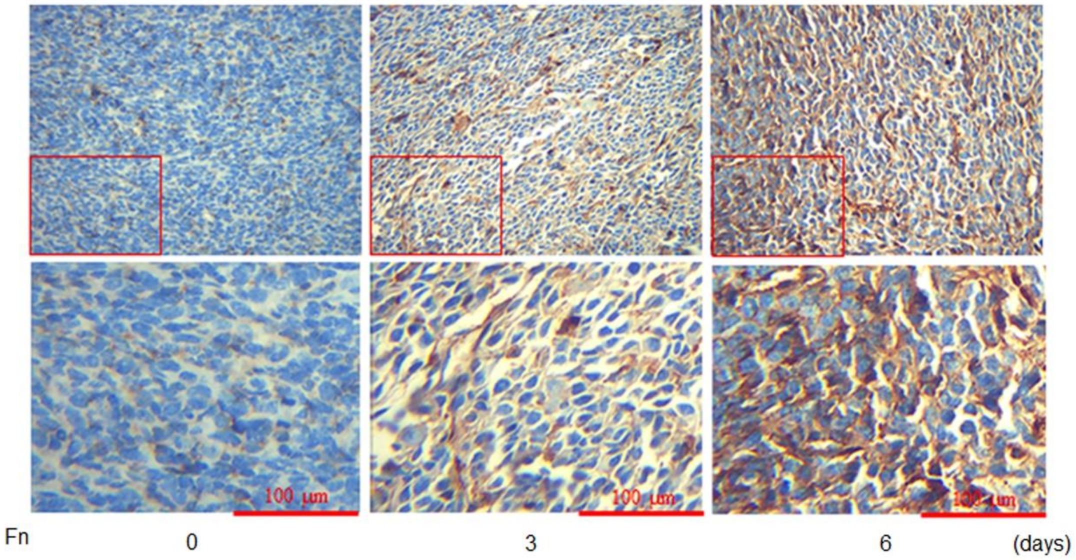

Fig. S7 *F. nucleatum* treatment enhanced PD-L1 expression in mouse tumor tissues. CT26.WT cells were subcutaneously injected into BALB/c mice. When the tumor volumes reached more than 100 mm^3, tumor-bearing mice were intratumorally injected with *F. nucleatum* (10^9 CFU) for 3 days or 6 days. The expression of PD-L1 (brown) was detected by IHC. Hematoxylin (blue) was used to visualize nuclei. The IHC images are representatives of different treatment groups.

Gao et al. Supplementary Figure 8

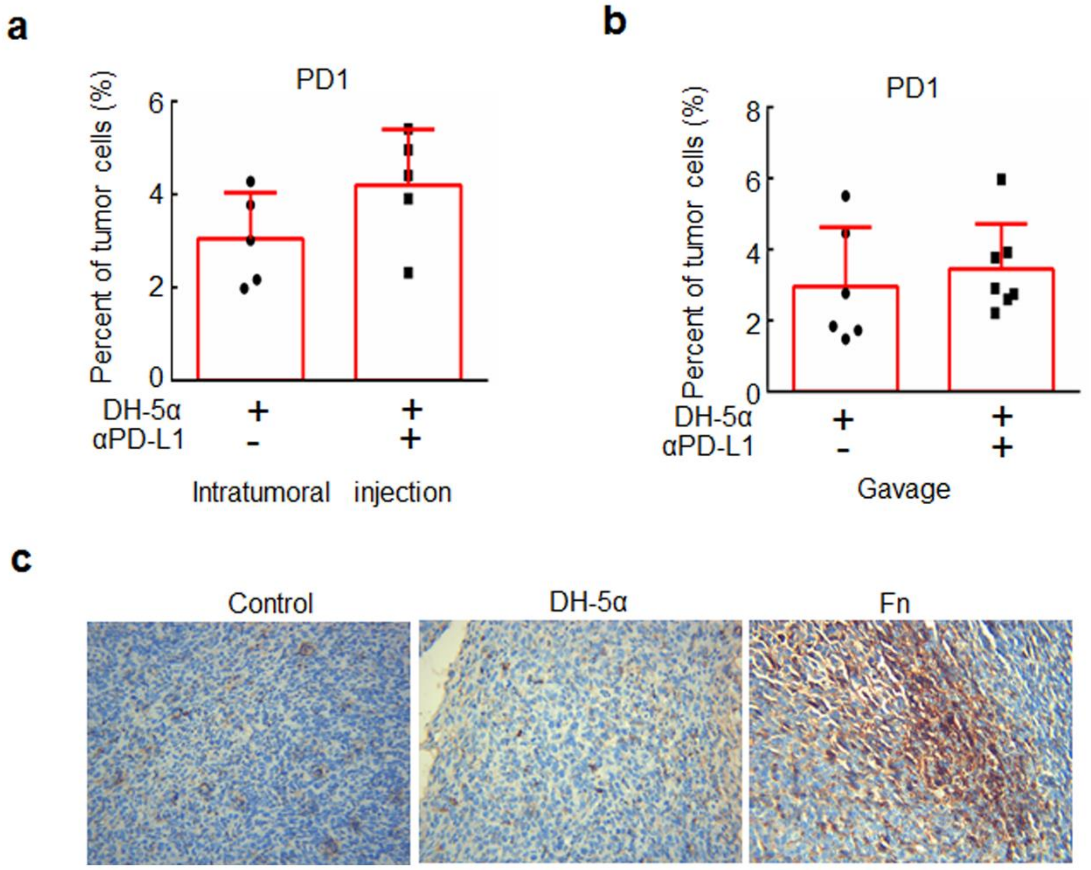

Fig. S8 DH-5α did not modulate the proportion of PD-1^+ T cells or PD-L1 expression during treatment with an anti-PD-L1 mAb. CT26.WT cells were subcutaneously injected into BALB/c mice. **a, c** Tumor-bearing mice were treated with DH-5α, *F. nucleatum* or PBS (control) by intratumoral injection or garage for 10 days. **a** Flow cytometry was used to detect the proportion of PD-1^+ cells in tumor tissue samples. Student's t-test. **c** The expression of PD-L1 (brown) detected by IHC. Hematoxylin (blue) was used to visualize nuclei. **b** Tumor-bearing mice were treated with DH-5α or PBS by garage for 8 days. Flow cytometry was used to detect the proportion of PD-1^+ cells in tumor tissue samples. Student's t-test.

Gao et al. Supplementary Figure 9

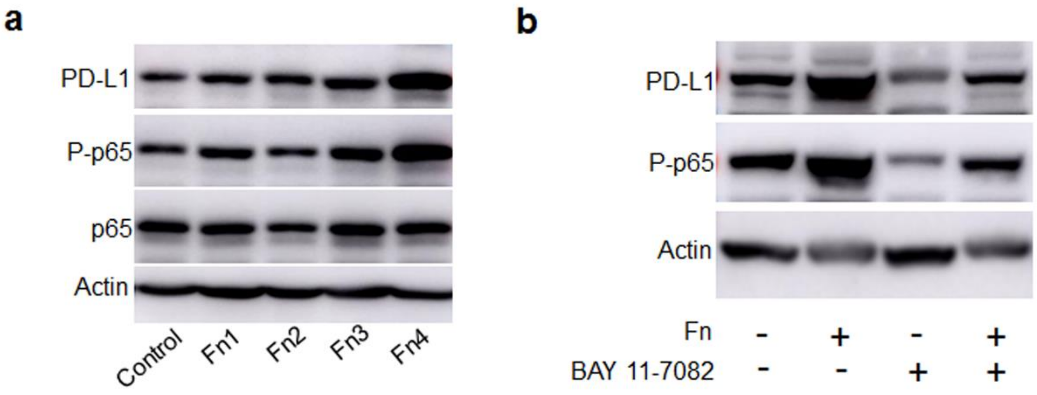

Fig. S9 *F. nucleatum* upregulated the expression of PD-L1 by phosphorylation of P65. **a** DLD1 cells were treated with different *F. nucleatum* isolates obtained from CRC patients, and the expression of the indicated proteins was detected. **b** Caco-2 cells were treated with Fn (1:1000) and/or 5 μM BAY 11-7082 for 24 hours. Protein expression was detected by Western blotting.

Gao et al. Supplementary Figure 10

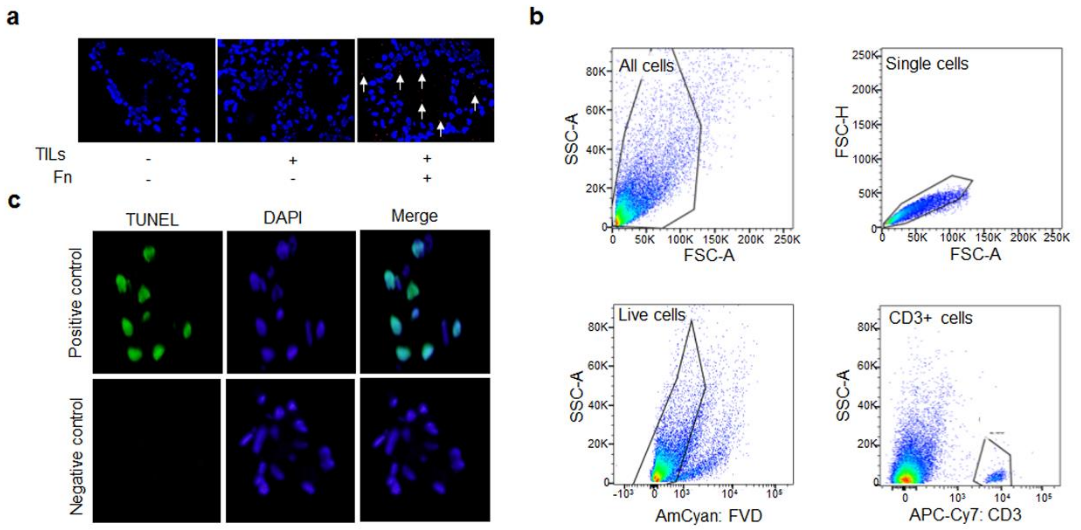

Fig. S10 Validation of tumor-derived organoid models of CRC. CRC organoids were mixed with TILs (10^5/well) and *F. nucleatum* (10^8 CFU) for one week. **a** *F. nucleatum* was detected in the CRC organoids with a FISH assay. The nuclei (blue) of cells in tumor tissue samples were stained with DAPI. The white arrows indicate positive staining (red) for *F. nucleatum*. **b** Flow cytometry was used to detect $CD3^+$ T cells in the CRC organoids. **c** The controls for the TUNEL assay are shown. The CRC organoids without treatment were used as negative controls. The CRC organoids with DNase I treatment (1500u/ml) for 30 min were used as negative controls.

Supplementary Table 1

Characteristics		No. of patients	Fn content High	Fn content Low	P value
Age (years)	≥ 65	11	7	4	0.3096
	< 65	16	7	9	
Gender	Male	17	10	7	0.3445
	Female	10	4	6	
Liver metastasis	Yes	14	9	5	0.1796
	No	13	5	8	
Lung metastasis	Yes	13	7	6	0.8416
	No	14	7	7	
Abdominal cavity metastasis	Yes	7	1	6	0.0208*
	No	20	13	7	
pelvic metastasis	Yes	4	2	2	0.936
	No	23	12	11	
Ovarian metastasis	Yes	4	2	2	0.936
	No	23	12	11	
Bone metastasis	Yes	4	2	2	0.936
	No	23	12	11	

Table S1. Correlations between the relative *F. nucleatum* levels in tumor tissues and clinicopathological characteristics of the patients with CRC. The Chi-square test was used for analysis.

Supplementary Table 2

**Characteristics**		**No. of patients**	**Fn**		**P value**
			**Yes**	**No**	
Age (years)	⩾65	15	11	4	0.8023
	＜65	23	16	7	
Gender	Male	28	21	7	0.3693
	Female	10	6	4	
Liver metastasis	Yes	23	15	8	0.326
	No	15	12	3	
Lung metastasis	Yes	21	14	7	0.5076
	No	17	13	4	
Abdominal cavity metastasis	Yes	9	8	1	0.1768
	No	29	19	10	
pelvic metastasis	Yes	4	2	2	0.3263
	No	34	25	9	
Ovarian metastasis	Yes	2	1	1	0.5
	No	36	26	10	
Bone metastasis	Yes	6	5	1	0.4698
	No	32	22	10	

Table S2. Correlations between the relative *F. nucleatum* levels in feces and clinicopathological characteristics of the patients with CRC. The Chi-square test was used for analysis.
